# Lipohypertrophy contributing to severe diabetic ketoacidosis in type 1 diabetes: An increasing problem in the context of the COVID‐19 pandemic?

**DOI:** 10.1002/ccr3.6517

**Published:** 2022-11-19

**Authors:** Emilia Imogen Smith, Andra Ciutac, Jamie C. Smith

**Affiliations:** ^1^ University of Manchester Manchester UK; ^2^ Department of Diabetes, Endocrinology & Metabolic Medicine Torbay Hospital Torquay UK

**Keywords:** diabetic ketoacidosis, lipohypertrophy, type 1 diabetes

## Abstract

A 57‐year‐old man with type 1 diabetes was admitted with diabetic ketoacidosis during the COVID‐19 pandemic. On examination, there was evidence of severe lipohypertrophy on his abdomen. Repeated injection of subcutaneous insulin into areas of lipohypertrophy was thought to have contributed to the development of ketoacidosis.

## Abstract 2

Lipohypertrophy is a complication of insulin injection therapy, leading to erratic systemic absorption of insulin and contributing to the development of diabetic ketoacidosis. Injection sites need to be examined carefully at diabetes annual reviews.

## 1 CASE

**FIGURE 1:**
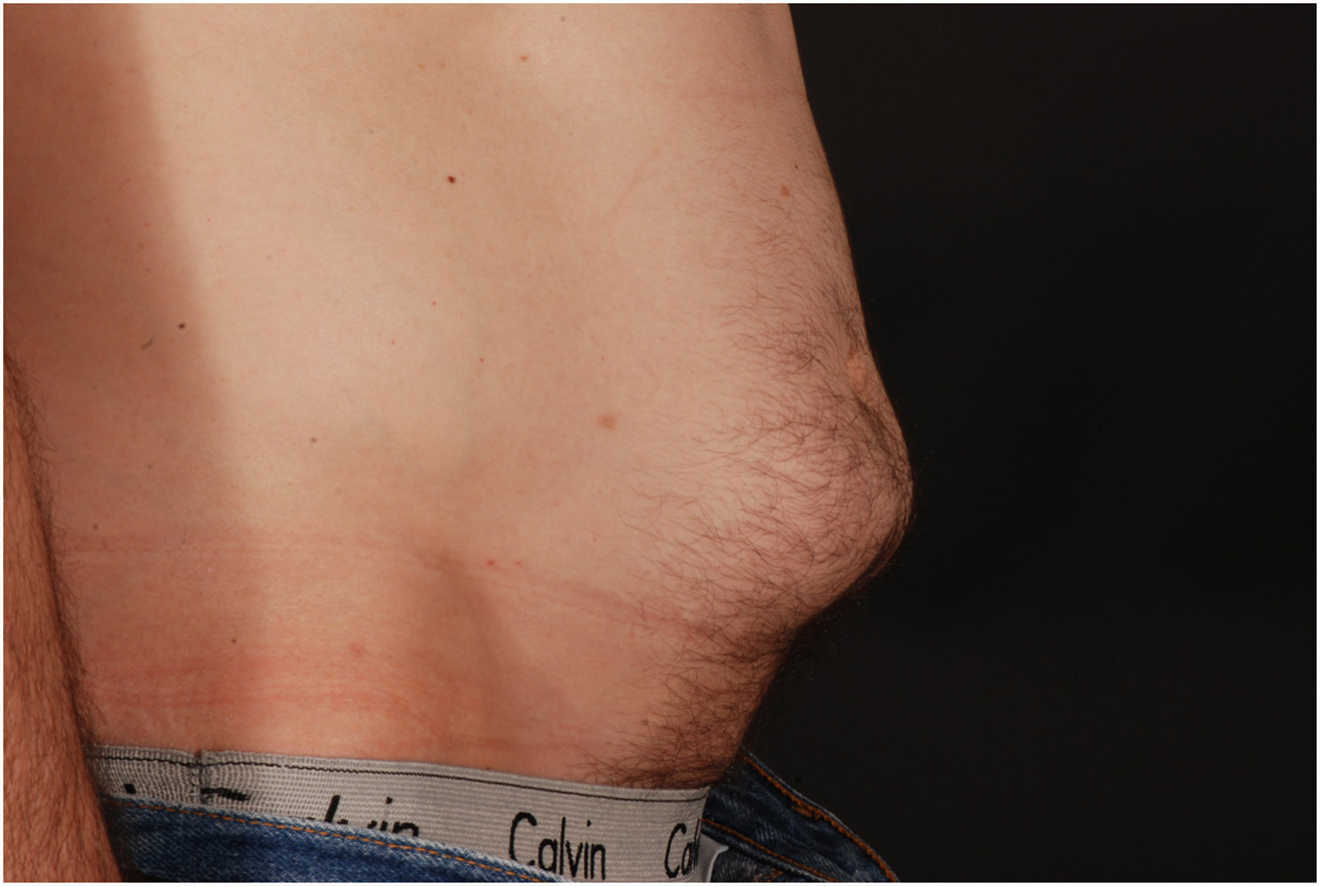
Areas of severe lipohypertrophy (lateral view), best seen in the upright posture, which corresponded to areas of subcutaneous insulin injection sites in a patient with longstanding type 1 diabetes admitted with diabetic ketoacidosis.

**FIGURE 2:**
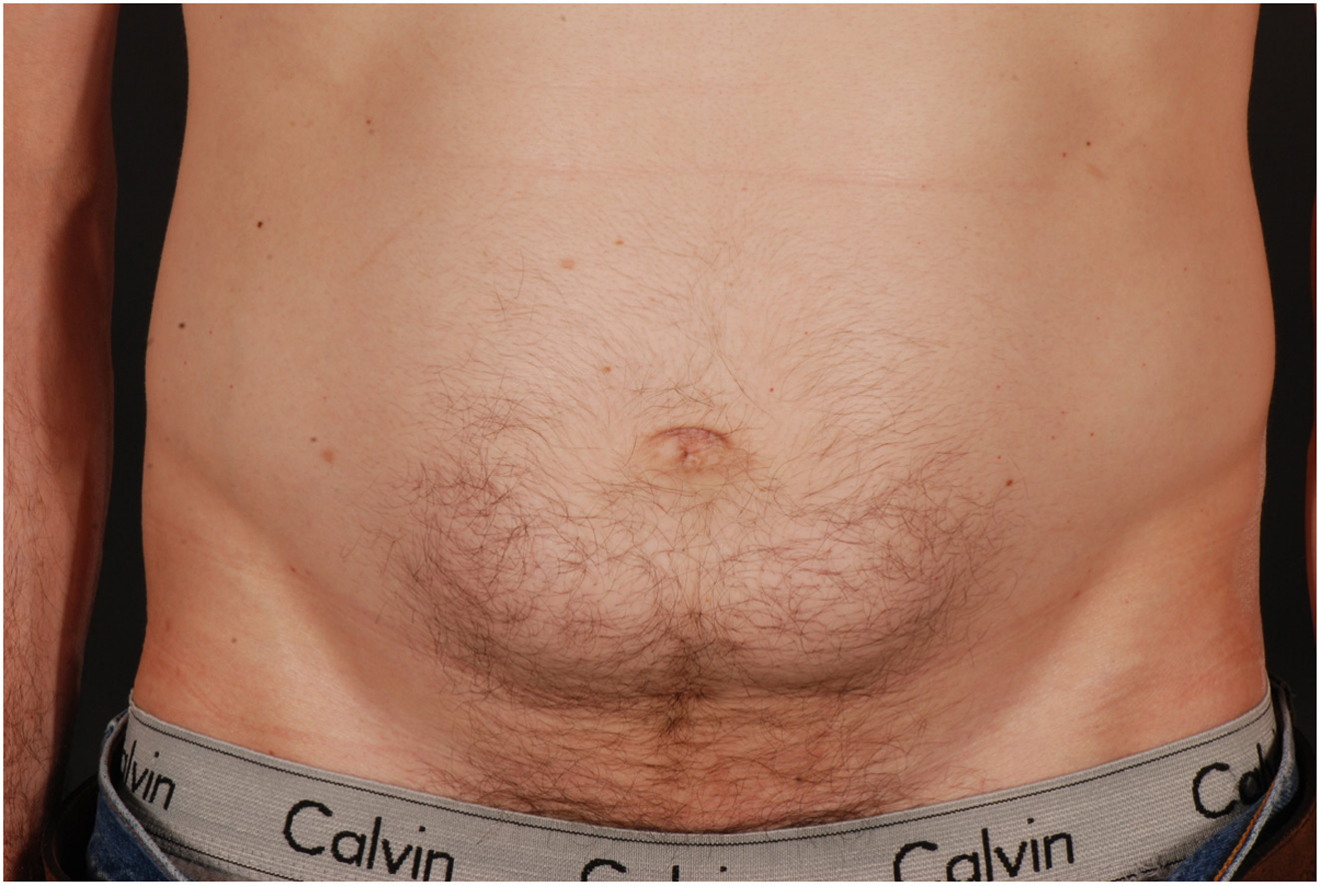
Areas of severe lipohypertrophy (anterior view), best seen in the upright posture, which corresponded to areas of subcutaneous insulin injection sites in a patient with longstanding type 1 diabetes admitted with diabetic ketoacidosis.

A 57‐year‐old man with type 1 diabetes was admitted with diabetic ketoacidosis during the COVID‐19 pandemic. He reported the administration of insulin on a regular basis into his abdomen but had noticed that blood glucose levels were rising despite increasing insulin doses. His abdomen was examined by a diabetes physician (Figures 1 and 2).

What do the figures indicate and how might this have contributed to the presentation?

**Answer:** Severe lipohypertrophy on both sides of the abdomen corresponding to insulin injection sites.

Lipohypertrophy is defined as the excessive proliferation of adipose tissue, commonly occurring at the site of repeated insulin injections. As a result, reduced systemic uptake of insulin occurs at the affected sites. Patients with this condition are more prone to developing hyperglycemic states. Alongside pain, bleeding, and bruising, lipohypertrophy is one of the most frequently encountered insulin site reactions.^1^ During the COVID‐19 pandemic, there has been less attention to this problem with the increasing use of virtual consultations and therefore a relative lack of face‐to‐face examinations to detect this clinically important problem. Healthcare professionals involved in diabetes care should be educated to be alert to this problem, and the checking of injection sites during the annual diabetes review should always be undertaken.

## AUTHOR CONTRIBUTIONS

ES and AC involved in literature search and preparation of the manuscript. JS involved in clinical management of the patient and concept of writing the manuscript, reviewed the manuscript, and made changes.

## CONFLICT OF INTEREST

None declared.

## CONSENT

Written informed consent was obtained from the patient to publish this report in accordance with the journal's patient consent policy.

## Data Availability

None.
